# Pregnancy rate and outcomes after uterine artery embolization for women: a systematic review and meta-analysis with trial sequential analysis

**DOI:** 10.3389/fmed.2023.1283279

**Published:** 2023-12-21

**Authors:** Xiaoli Yan, Le Zhou, Guolin He, Xinghui Liu

**Affiliations:** ^1^Department of Obstetrics and Gynecology, West China Second University Hospital, Sichuan University, Chengdu, China; ^2^Department of Gynecology and Obstetrics, Southwest Hospital, Third Military Medical University (Army Medical University), Chongqing, China; ^3^Laboratory of the Key Perinatal Diseases, Key Laboratory of Birth Defects and Related Diseases of Women and Children, Ministry of Education, West China Second University Hospital, Sichuan University, Chengdu, China

**Keywords:** uterine artery embolization, uterine fibroids, postpartum hemorrhage, cesarean scar pregnancy, pregnancy outcomes

## Abstract

**Objective:**

The assessment of the relative impacts of uterine artery embolization (UAE) treatment for female patients is a critical field that informs clinical decisions, yet there is a noticeable scarcity of high-quality, long-term comparative studies. This meta-analysis aimed to focus on the pregnancy rate and outcomes in female patients following UAE and to conduct subgroup analyses based on different patient populations or various control treatments.

**Methods:**

A systematic literature search was conducted on 2 August 2023 through the Web of Science, PubMed, Embase, and the Cochrane Library of Clinical Trials for all potential studies. Relative risks (RRs) with 95% confidence intervals (CIs) were applied to compare pregnancy rates and outcomes between the UAE group and the control group. Heterogeneity was evaluated statistically by using the chi-square-based Cochran’s Q test and Higgins I^2^ statistics, and 95% prediction interval (PI). Software R 4.3.1 and Stata 12.0 were used for meta-analysis. The trial sequential analysis (TSA) was performed with TSA v0.9.5.10 Beta software.

**Results:**

A total of 15 eligible studies (11 cohort studies, 3 randomized controlled trials, and 1 non-randomized clinical trial) were included in this meta-analysis. The overall results revealed that UAE significantly decreased postoperative pregnancy rate [RR (95% CI): 0.721 (0.531–0.979), 95% PI: 0.248–2.097] and was associated with an increased risk of postoperative PPH [RR (95% CI): 3.182 (1.319–7.675), 95% PI: 0.474–22.089]. Analysis grouped by population indicated that UAE decreased the risk of preterm delivery [RR (95% CI): 0.326 (0.128–0.831), *p* = 0.019] and cesarean section [RR (95% CI): 0.693 (0.481–0.999), *p* = 0.050] and increased the risk of placenta previa [RR (95% CI): 8.739 (1.580–48.341), *p* = 0.013] in patients with UFs, CSP, and PPH, respectively. When compared with myomectomy, HIFU, and non-use of UAE, UAE treatment was associated with the reduced risks of preterm delivery [RR (95% CI): 0.296 (0.106–0.826)] and cesarean section [(95% CI): 0.693 (0.481–0.999), *p* = 0.050] and increased placenta previa risk [RR (95% CI): 10.682 (6.859–16.636)], respectively.

**Conclusion:**

UAE treatment was associated with a lower postoperative pregnancy rate and increased risk of PPH. Subgroup analysis suggested that UAE was shown to decrease the risk of preterm delivery and cesarean section and increase placenta previa risk.

**Systematic review registration:**https://www.crd.york.ac.uk/prospero/, Identifier CRD42023448257.

## 1 Introduction

Uterine artery embolization (UAE) represents a minimally invasive intervention frequently applied in the management of both acute and chronic genital hemorrhage stemming from a spectrum of obstetric and gynecological conditions (1). Optimal candidates for UAE encompass individuals afflicted with symptomatic fibroids, who express a desire for uterine preservation and/or seek alternatives to surgical procedures (2). Recent years have seen the extensive use of UAE in addressing conditions, such as uterine fibroids (UFs), postpartum hemorrhage (PPH), and cesarean scar pregnancy (CSP) (3–5). UAE has demonstrated efficacy in symptom improvement with a low incidence of major complications. However, certain studies have reported potential adverse effects, including postembolization syndrome and subclinical impairment of ovarian function (6, 7). Furthermore, radiation exposure during the procedure may pose a risk to the genital system, with particular concern for the ovaries and endometrium, potentially leading to future fertility issues (8).

The American College of Obstetricians and Gynecologists advises a cautious approach to the application of UAE for patients intending to conceive, due to the insufficiently explored effect of UAE on pregnancy (9). Conversely, guidelines from the Society of Interventional Radiology (SIR) advocate for the consideration of UAE as a viable option for those targeting subsequent fertility, contingent upon individual patient preferences and specific case nuances (10). Previous systematic reviews and meta-analyses have reported the fertility results and pregnancy outcomes after UAE. For example, a systematic review published by Li et al. revealed that the pregnancy rate after myomectomy (43%) was higher than 18% after high-intensity focused ultrasound (HIFU), and the latter was significantly higher than that after UAE (8%) (11). The study by Matsuzaki et al. demonstrated that women who had previously undergone UAE faced a heightened risk of PPH in subsequent pregnancies (12).

The assessment of the relative impacts of minimally invasive treatments for female patients is a critical field that informs clinical decisions, yet there is a noticeable scarcity of high-quality, long-term comparative studies. Understanding the potential risks associated with maternal and obstetric outcomes in pregnancies subsequent to UAE could prove instrumental in its antenatal diagnosis and treatment involving multidisciplinary care (13, 14). Therefore, we have implemented a current systematic review and meta-analysis on pregnancy rate and outcomes following UAE treatments for female patients and further performed subgroup analyses by patient populations (such as UFs, CSP, and PPH) or control treatments (myomectomy, HIFU, or other treatments) to find out whether a safe pregnancy is possible after UAE.

## 2 Materials and methods

The Preferred Reporting Items for Systematic Reviews and Meta-Analyses (PRISMA) guidelines were followed in writing the current study (15). The protocol for this review has been prospectively registered in the PROSPERO database (CRD42023448257).

### 2.1 Search strategy

A systematic literature search was conducted on 2 August 2023 through the Web of Science, PubMed, Embase, and the Cochrane Library of Clinical Trials for all potential studies. Search filters were set to articles published from inception to 2 July 2023 and in English. The following search items were used: (“uterine artery embolization” OR “UAE”) AND (“pregnancy outcome” OR “outcomes” OR “pregnancies” OR “gestation” OR “reproduction”) AND (“cohort study” OR “retrospective study” OR “randomized clinical trial” OR “controlled clinical trial”). The detailed search strategy is provided in Supplementary Table S1. In addition, the reference lists of qualified articles and related reviews were screened to ensure complete study capture.

### 2.2 Study selection

The following were among the inclusion criteria: (i) cohort studies, randomized controlled trials (RCTs), or non-randomized controlled studies; (ii) the UAE group and control group were considered in the study design; and (iii) study results involving at least one pregnancy outcome of interest for the present analysis. Exclusion criteria were as follows: (i) case–control studies; (ii) single-center studies without a control group; (iii) studies with duplicate original data; and (iv) systematic reviews, meta-analyses, conference abstracts, and case reports.

### 2.3 Data extraction

Two researchers independently extracted all information and data from the included studies. The disagreements were resolved by the third reviewer through cross-discussion or consultation. Data were extracted using separate Excel spreadsheets. We extracted the following information from included studies using the predesigned data-collection form: first author’s name and publication year, study design, country, patient’s age, disease type, sample size and treatment of exposure group and control group, follow-up time, and outcomes (including primary outcomes: pregnancy rate, spontaneous abortion, and live birth; secondary outcomes: ectopic pregnancy, cesarean section, preterm delivery, postpartum hemorrhage, and placenta previa).

### 2.4 Quality assessment

The quality of the included cohort studies was assessed according to the Newcastle–Ottawa Scale (NOS) (16), consisting of selection of subjects, comparability of groups, and assessment of outcome. The quality of each cohort study was considered as low (0–3 score out of 9), moderate (4–6 score out of 9), or high (7–9 score out of 9) (17). The quality of RCTs was evaluated using the modified Jadad scale (18), consisting of randomization, randomization concealment, double-blind, and withdrawals and dropouts. A score of 0–3 or 4–7 out of 7 was considered a low-quality or high-quality study, respectively. Two reviewers conducted a risk-of-bias assessment, and any inconsistencies were handled by the third reviewer.

### 2.5 Statistical analysis

Software R 4.3.1 and Stata 12.0 (Stata Corp. College Station, Texas, United States) were used for all analyses. Relative risks (RRs) with 95% confidence intervals (CIs) were applied to compare the pregnancy rates and outcomes between the UAE group and the control group. Heterogeneity was evaluated statistically by using the chi-square-based Cochran’s Q test and Higgins I^2^ statistics, and 95% prediction interval (PI) (19, 20). Results with I^2^ > 50% or *p* < 0.10 were considered to exhibit significant heterogeneity, and a random-effects model was then applied; otherwise, the fixed-effects model was adopted (21). Subgroup analyses were applied to explore the potential sources of heterogeneity. We also performed a sensitivity analysis to test the stability of the present analysis. Publication bias was calculated using visual interpretation of funnel plots and Begg’s and Egger’s tests (22, 23). If any publication bias existed, it was quantitatively adjusted by the trim-and-fill method (24).

### 2.6 Trial sequential analysis

We conducted a trial sequential analysis (TSA) to assess the strength of evidence and adjust for potential errors (25). The TSA was performed with TSA v0.9.5.10 Beta software^1^ to calculate the required information size (RIS) and trial sequential monitoring boundaries. We built O′ Brien-Fleming α-spending boundaries by setting a type I error of 5% with a power of 80%, which were two-sided values. If the cumulative Z-curve crossed the RIS boundary or trial sequential monitoring boundary, further research studies were unnecessary, and firm evidence was obtained to accept or refute the intervention effect.

## 3 Results

### 3.1 Study selection procedure

**Figure 1:**
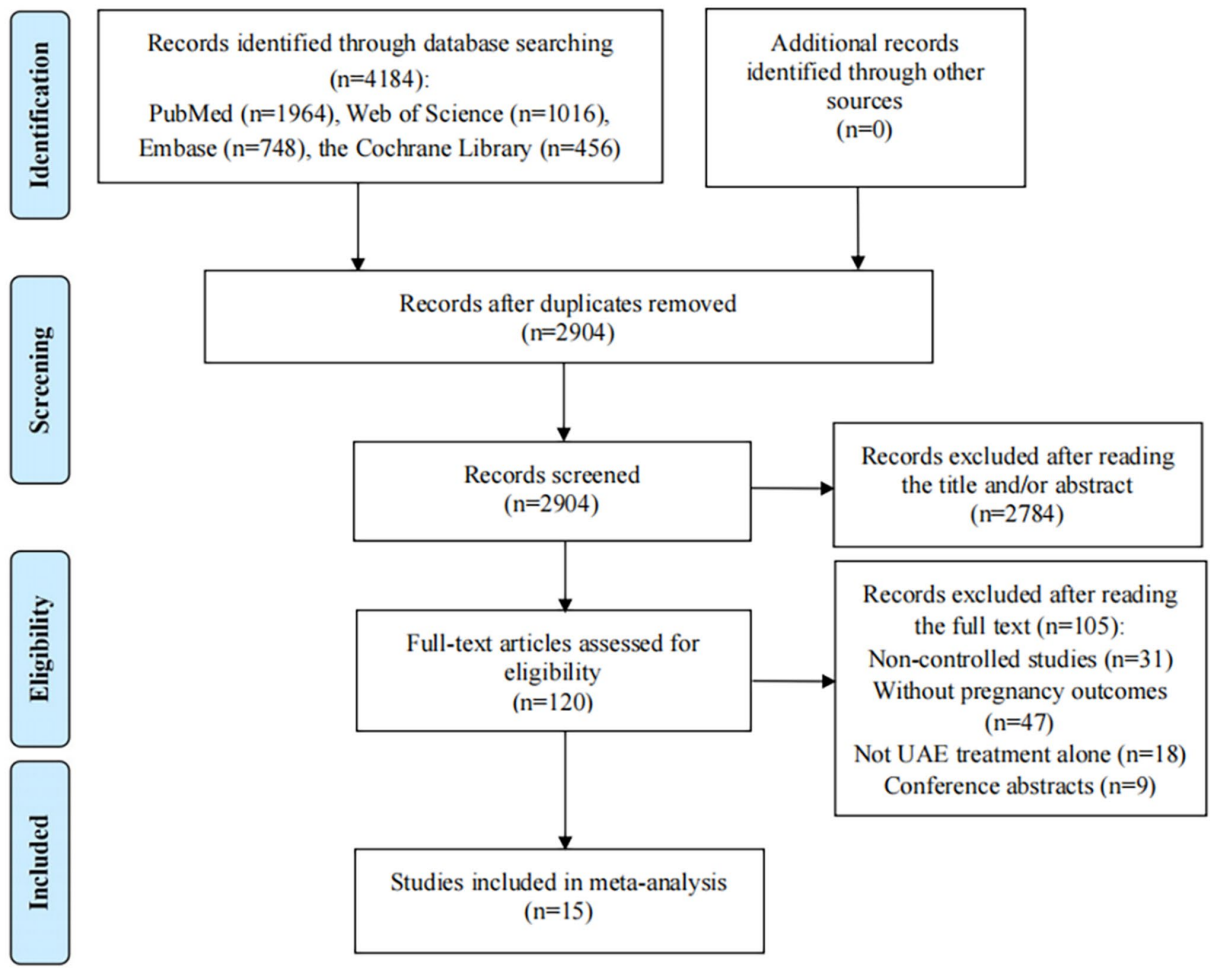
Flow diagram of the process of selection of articles.

The initial database search in Web of Science, PubMed, Embase, and the Cochrane Library of Clinical Trials identified 4,184 records; after 1,280 duplicates were excluded, 2,904 records remained. Then, title/abstract screening was conducted on 2,904 articles, during which 2,784 articles were eliminated due to irrelevancy. After reading the remaining 120 articles for full-text review, 105 articles did not meet our inclusion/exclusion criteria: 31 were non-controlled studies; 47 studies did not provide pregnancy rates or outcomes after UAE treatment; interventions in 18 studies were not UAE treatment alone; and 9 studies were abstracts. Finally, 15 studies were included in this meta-analysis, including 11 cohort studies and 4 clinical trials (Figure 1) (26–40).

### 3.2 Study characteristics and quality assessment

The features of the included studies and research participants are provided in Table 1. The included studies contained 11 cohort studies, 3 RCTs, and 1 non-randomized clinical trial. Eligible studies were published between 2008 and 2023 and performed in France, China, the USA, the Czech Republic, the UK, Japan, Korea, and Germany. A total of 964,398 women with postpartum hemorrhage, cesarean scar pregnancy, uterine fibroid, retained products of conception, or cervical pregnancy were included in the UAE group and the control group. The pregnancy rate was calculated as a proportion of patients intending to conceive where this information was available, or as a proportion of total patients recruited in the exposure or control group where the above information was not available. The remaining rates of pregnancy outcomes were calculated as a proportion of pregnant patients or total patients recruited in the exposure or the control group. The total scores of the 10 cohort studies and 3 RCTs ranged from 7 to 9 and 4 to 7, respectively, indicating a low risk of bias. One cohort study and one clinical trial were assessed as low quality because the study design had not been described in detail (Supplementary Table S2).

**Table 1:** Characteristics of eligible studies.

Author/year	Study design	Country/region	Age (years)	Population	Exposure group		Control group		Follow-up duration	Outcomes
					Sample size	Treatment	Sample size	Treatment		
Hardeman et al., 2010 (26)	RCS	France	Median (IQR): 34.3 (19–44)	PPH patients	53	UAE	106	Without UAE	E: 82 months (maximum) C: 83 months (maximum)	1
Chen et al., 2015 (27)	RCS	China	E: 32 ± 3.3 C: 32.7 ± 4.4	CSP patients	38	UAE	90	Transvaginal debridement and repair surgery	≥12 months	1
Borah et al., 2017 (28)	RCS	USA	43.2 ± 6.0	UF patients	4,186	UAE	19,965	Myomectomy	3.4 months (mean)	1, 2, 4, 5, 6, 7
Mara et al., 2008 (29)	RCT	Czech Republic	E: 32.4 C: 32.0	UF patients	58	UAE	63	Myomectomy	24.9 months (mean)	1, 2, 4, 5, 6, 7
Edwards et al., 2007 (30)	RCT	UK	E: 43.6 ± 5.5 C: 43.3 ± 7.1	UF patients	106	UAE	51	Myomectomy	32 months (median)	1, 2, 3, 5
Imafuku et al., 2020 (31)	RCS	Japan	Median (range): E: 30.5 (26–38) C: 32.0 (21–41)	PPH patients	81	UAE	206	Without UAE	NR	1, 5, 6, 7, 8
Chen et al., 2019 (32)	RCS	China	NR	CSP patients	67	UAE	68	HIFU	1–10 years	1, 2, 5, 6
Daniels et al., 2021 (33)	RCT	UK	E: 40.2 ± 6.55 C: 42.7 ± 6.40	UF patients	127	UAE	127	Myomectomy	4 years	1, 3
Ohmaru-Nakanishi et al., 2019 (34)	RCS	Japan	E: 32.9 ± 0.9 C: 32.3 ± 1.3	Patients with RPOC	32	UAE	25	Without UAE	NR	1, 5, 6, 7, 8
Wang et al., 2023 (35)	RCS	China	E: 31.12 ± 5.39 C: 31.39 ± 5.02	CSP patients	118	UAE	154	HIFU-a	30 months (mean)	1, 2, 5
Jitsumori et al., 2020 (36)	RCS	Japan	E: 35.0 ± 4.3 C: 33.7 ± 5.4	PPH patients	16	UAE	3,139	Without UAE	NR	6
Cho et al., 2017 (37)	RCS	Korea	E: 32.51 ± 3.12 C: 31.07 ± 3.53	PPH patients	1,222	UAE	933,987	Without UAE	NR	1, 5, 8
Froeling et al., 2013 (38)	RCS	Germany	Median (range): E: 42.7 (33.6–52.2) C: 36.2 (29.2–41.0)	UF patients	41	UAE	36	MR-g HIFU	E: 61.9 months (median) C: 60.7 months (median)	1, 3
Li et al., 2022 (39)	RCS	China	E: 33.3 ± 1.12 C: 33.2 ± 1.75	Patients with CP	25	UAE	11	HIFU-a	41.9 months (mean)	1
Mara et al., 2012 (40)	Prospective non-randomized trial	Czech Republic	E: 33.1 ± 3.7 C: 34.9 ± 4.0	UF patients	100	UAE	100	LUAO	E: 45.5 months (mean) C: 40.4 months (mean)	1, 4, 5, 6, 8

RCS, retrospective cohort study; RCT, randomized controlled trial; IQR, interquartile range; UAE, uterine artery embolization; PPH, postpartum hemorrhage; CSP, cesarean scar pregnancy; UF, uterine fibroid; RPOC, retained products of conception; CP, cervical pregnancy; HIFU, high-intensity focused ultrasound; NR, not reported; MR-g HIFU, magnetic resonance-guided high-intensity focused ultrasound; HIFU-a, high-intensity focused ultrasound ablation; LUAO, laparoscopic uterine artery occlusion; 1, pregnancy rate; 2, spontaneous abortion; 3, live birth; 4, ectopic pregnancy; 5, cesarean section; 6, preterm delivery; 7, postpartum hemorrhage; 8, placenta previa.

### 3.3 Pooled effect of primary outcomes after UAE

**Figure 2:**
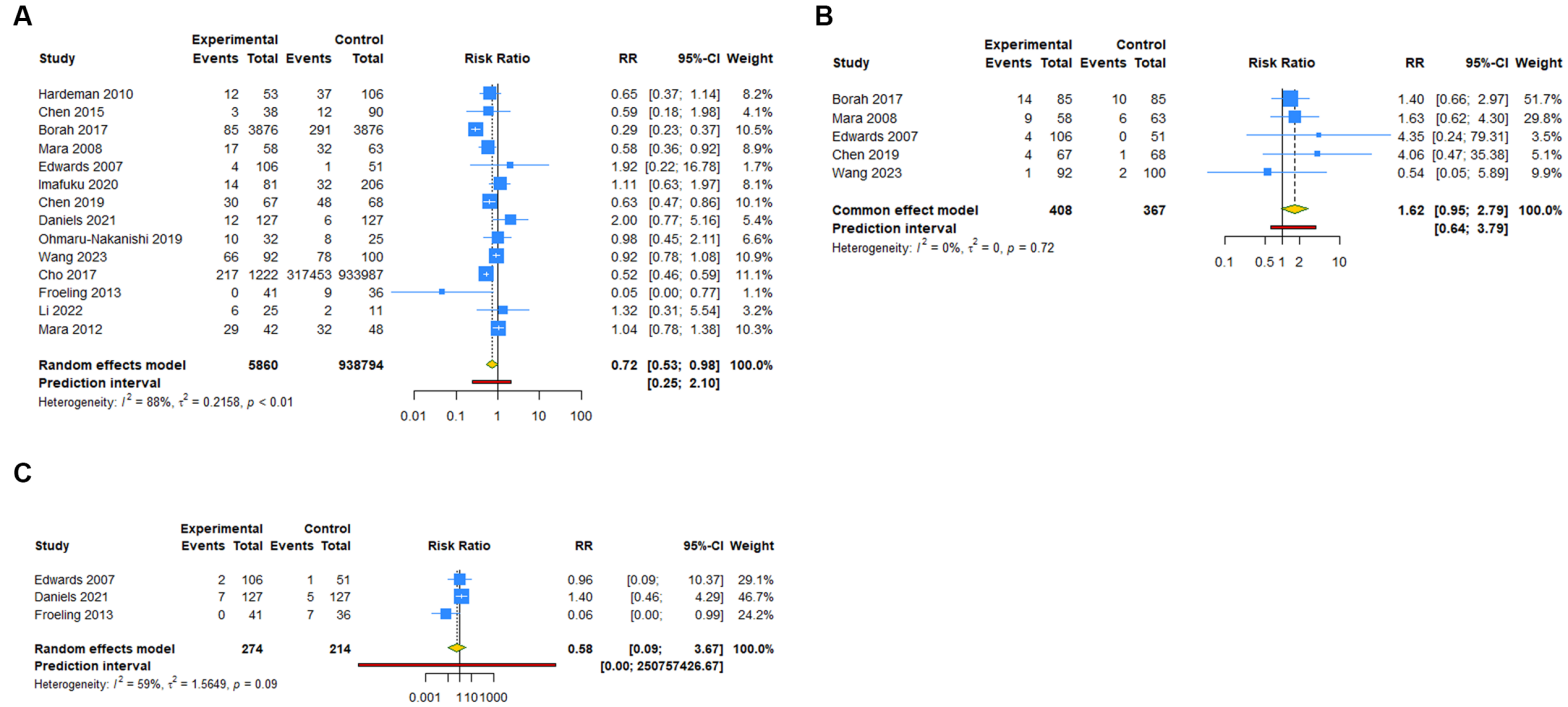
Forest plot of primary outcomes after UAE. **(A)** Pregnancy rate. **(B)** Spontaneous abortion. **(C)** Live birth.

**Figure 3:**
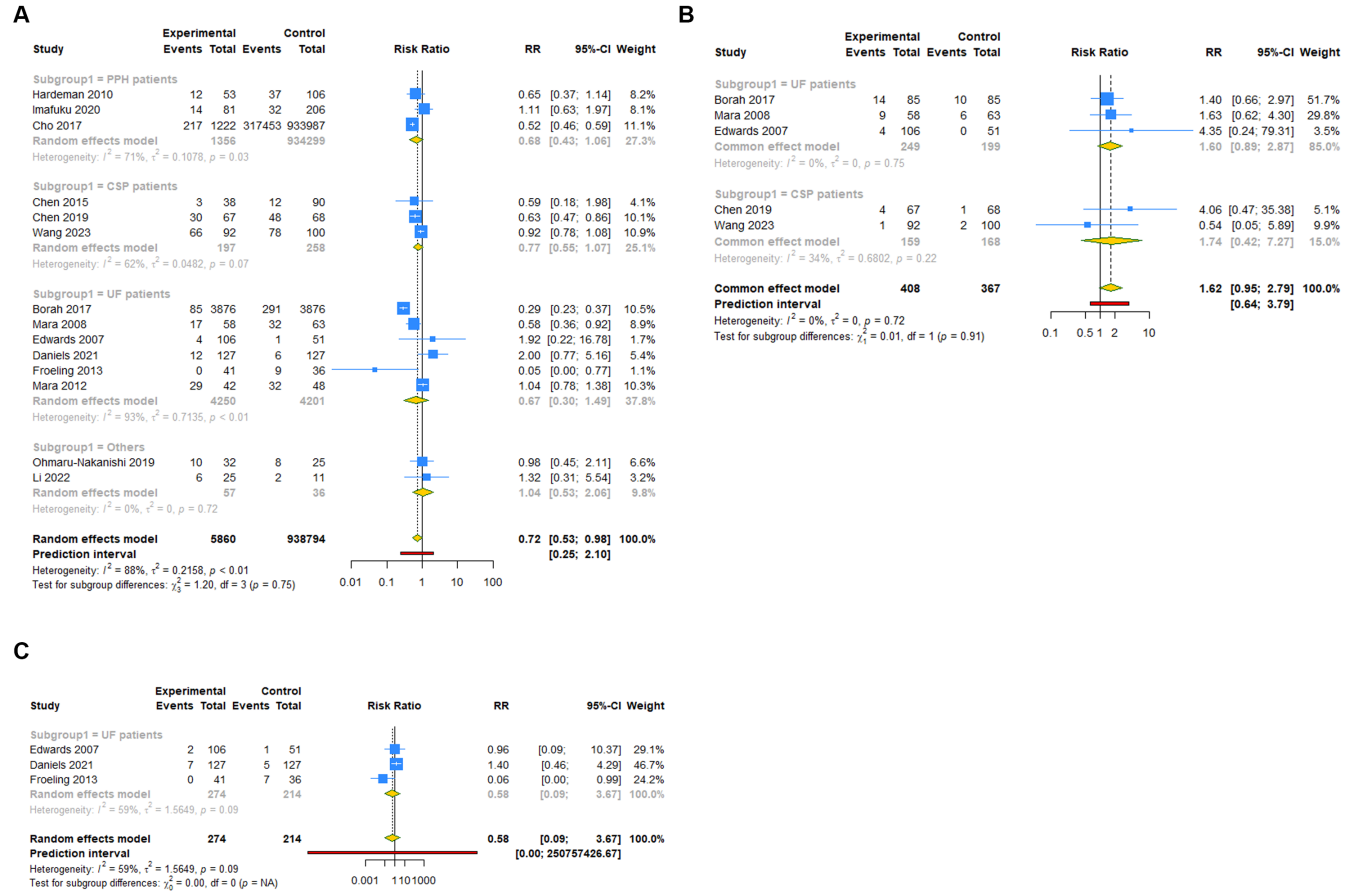
Subgroup analysis (Subgroup 1) of primary outcomes after UAE. **(A)** Pregnancy rate. **(B)** Spontaneous abortion. **(C)** Live birth.

**Figure 4:**
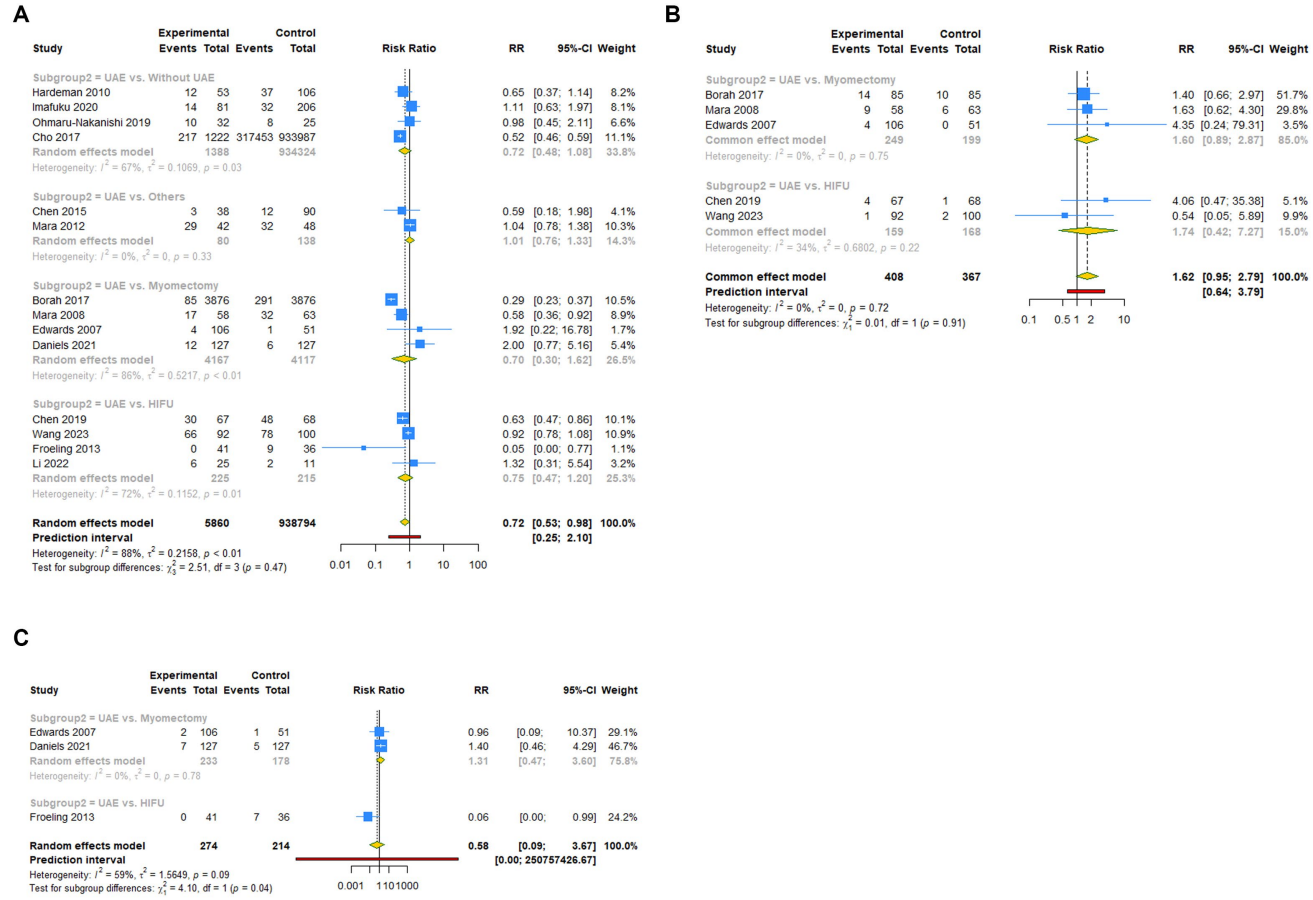
Subgroup analysis (Subgroup 2) of primary outcomes after UAE. **(A)** Pregnancy rate. **(B)** Spontaneous abortion. **(C)** Live birth.

A total of 14 studies reported pregnancy rate as an outcome measure. The pooled results from the random-effects model revealed that UAE significantly decreased postoperative pregnancy rate in female patients [RR (95% CI): 0.721 (0.531–0.979), 95% PI: 0.248–2.097; I^2^ = 88.4%, Tau^2^ = 0.2158; Table 2; Figure 2A]. Subgroup analysis showed that UAE treatment did not significantly affect the pregnancy rate in patients with PPH [RR (95% CI): 0.678 (0.434–1.058), *p* = 0.087], CSP [RR (95% CI): 0.769 (0.550–1.075), *p* = 0.124], UF [RR (95% CI): 0.669 (0.300–1.493), *p* = 0.327], or others [RR (95% CI): 1.058 (0.536–2.086), *p* = 0.872; Table 2; Figure 3A]. Meanwhile, compared with patients treated without UAE [RR (95% CI): 0.720 (0.480–1.079), *p* = 0.112] or with myomectomy [RR (95% CI): 0.701 (0.304–1.618), *p* = 0.405), HIFU (RR (95% CI): 0.752 (0.470–1.205), *p* = 0.236], or others [RR (95% CI): 0.950 (0.703–1.285), *p* = 0.741] in the control group, UAE did not decrease the postoperative pregnancy rate (Table 2; Figure 4A).

**Table 2:** Pooled effect and subgroup analysis of primary pregnancy outcomes after uterine artery embolization for women.

Outcomes and subgroups	Number of study	Meta-analysis				Heterogeneity	
		RR	95% CI	*p*-value	95% PI	I^2^, Tau^2^	*P*-value
**Pregnancy rate**							
Overall	14	0.721	0.531–0.979	0.036	0.248–2.097	88.4%, 0.2158	<0.001
**Subgrouped by the origin of participants (Subgroup 1)**							
PPH patients	3	0.678	0.434–1.058	0.087	0.004–108.027	70.7%, 0.1078	0.033
CSP patients	3	0.769	0.550–1.075	0.124	0.022–26.375	61.9%, 0.0482	0.072
UF patients	6	0.669	0.300–1.493	0.327	0.049–9.065	92.5%, 0.7135	<0.001
Others	2	1.058	0.536–2.086	0.872	-	0%, 0	0.715
**Subgrouped by the treatment of the control group (Subgroup 2)**							
UAE vs. Without UAE	4	0.720	0.480–1.079	0.112	0.136–3.802	66.8%, 0.1069	0.029
UAE vs. Myomectomy	4	0.701	0.304–1.618	0.405	0.019–25.903	86.2%, 0.5217	<0.001
UAE vs. HIFU	4	0.752	0.470–1.205	0.236	0.126–4.503	72.0%, 0.1152	0.013
UAE vs. Others	2	0.950	0.703–1.285	0.741	-	0%, 0	0.332
**Spontaneous abortion**							
Overall	5	1.623	0.946–2.786	0.079	0.640–3.792	0%, 0	0.719
**Subgrouped by the origin of participants (Subgroup 1)**							
CSP patients	2	1.743	0.418–7.271	0.446	-	33.5%, 0.6802	0.220
UF patients	3	1.602	0.894–2.870	0.113	0.035–67.773	0%, 0	0.749
**Subgrouped by the treatment of the control group (Subgroup 2)**							
UAE vs. Myomectomy	3	1.602	0.894–2.870	0.113	0.035–67.773	0%, 0	0.749
UAE vs. HIFU	2	1.743	0.418–7.271	0.446	-	33.5%, 0.6802	0.220
**Live birth**							
Overall	3	0.582	0.092–3.673	0.565	-	59.0%, 1.5649	0.087
**Subgrouped by the origin of participants (Subgroup 1)**							
UF patients	3	0.582	0.092–3.673	0.565	-	59.0%, 1.5649	0.087
**Subgrouped by the treatment of the control group (Subgroup 2)**							
UAE vs. Myomectomy	2	1.307	0.475–2.593	0.604	-	0%, 0	0.780
UAE vs. HIFU	1	0.059	0.004–0.994	0.050			

Regarding spontaneous abortion, a total of 5 studies reported the outcomes. Pooled results from the fixed-effects model indicated that UAE seems to increase the rate of spontaneous abortion in total population [RR (95% CI): 1.623 (0.946–2.786), 95% PI: 0.640–3.792; I^2^ = 0, Tau^2^ = 0], or CSP [RR (95% CI): 1.743 (0.418–7.271), *p* = 0.446] and UF [RR (95% CI): 1.602 (0.894–2.870), *p* = 0.113] patients compared with the control, but without statistical significance (Table 2; Figure 2B, Figure 3B). Analysis grouped by the treatment of the control group showed that UAE treatment did not significantly increase the postoperative spontaneous abortion rate compared with myomectomy [RR (95% CI): 1.602 (0.894–2.870), *p* = 0.113] or HIFU [RR (95% CI): 1.743 (0.418–7.271), *p* = 0.446; Table 2; Figure 4B].

The live birth rate was evaluated in 3 studies, all of which reported UF patients. The overall and subgroup analysis (subgroup 1) revealed that UAE treatment did not reduce the live birth rate among UF patients [RR (95% CI): 0.582 (0.092–3.673); I^2^ = 59.0%, Tau^2^ = 1.5649; Table 2; Figures 2C, 3C]. Analysis grouped by subgroup 2 indicated that compared with myomectomy, UAE seems to increase the rate of live birth, but without statistical significance [RR (95% CI): 1.307 (0.475–2.593), *p* = 0.604; Table 2; Figure 4C].

### 3.4 Pooled effect of secondary outcomes after UAE

Three studies reported ectopic pregnancy and UF patients. The overall and subgroup analysis (subgroup 1) revealed that UAE treatment did not increase the risk of ectopic pregnancy among UF patients [RR (95% CI): 1.218 (0.420–3.533); I^2^ = 0, Tau^2^ = 0]. Analysis grouped by subgroup 2 indicated that compared with myomectomy, UAE seems to increase the risk of ectopic pregnancy, but without statistical significance [RR (95% CI): 1.017 (0.302–3.422), *p* = 0.979; Supplementary Table S3; Supplementary Figure S1].

Nine studies reported cesarean section as a secondary pregnancy outcome measure. The overall results revealed that UAE treatment did not significantly decrease the cesarean section risk [RR (95% CI): 0.945 (0.664–1.345), 95% PI: 0.326–2.741; I^2^ = 77.1%, Tau^2^ = 0.1703]. Subgroup analysis showed that UAE did not significantly affect the incidence of cesarean section in patients with UF [RR (95% CI): 0.881 (0.494–1.570), *p* = 0.667] and PPH [RR (95% CI): 1.231 (0.706–2.144), *p* = 0.464]. Meanwhile, compared with patients treated with myomectomy [RR (95% CI): 0.574 (0.184–1.793), *p* = 0.340] or without UAE [RR (95% CI): 1.297 (0.824–2.040), *p* = 0.261] in the control group, UAE had no effect on the risk of cesarean section. However, significantly reduced cesarean section risk after UAE was found in CSP patients, and when compared with HIFU, UAE decreased the risk of cesarean section [RR (95% CI): 0.693 (0.481–0.999), *p* = 0.050; Supplementary Table S3; Supplementary Figure S2].

Regarding preterm delivery, a total of 7 studies reported the outcomes. Pooled results indicated that UAE seems to decrease the risk of preterm delivery compared with the control, but without statistical significance [RR (95% CI): 0.632 (0.356–1.121), 95% PI: 0.326–1.609; I^2^ = 0, Tau^2^ = 0]. Analysis grouped by subgroup 1 revealed that UAE treatment significantly reduced the risk of preterm delivery in UF patients [RR (95% CI): 0.326 (0.128–0.831), *p* = 0.019]. Analysis grouped by the treatment of the control group showed that compared with myomectomy, UAE treatment decreased the incidence of postoperative preterm delivery [RR (95% CI): 0.296 (0.106–0.826), *p* = 0.020; Supplementary Table S3; Supplementary Figure S3].

Four studies reported PPH. The overall analysis revealed that UAE treatment was associated with a significantly increased risk of postoperative PPH [RR (95% CI): 3.182 (1.319–7.675), 95% PI: 0.474–22.089; I^2^ = 0, Tau^2^ = 0]. No significant relationship was found between UAE treatment and PPH in subgroups that included 2 studies (all *p* > 0.05; Supplementary Table S3; Supplementary Figure S4).

A total of 4 studies reported regarding placenta previa. The overall results showed that there was no significant correlation between UAE treatment and the risk of postoperative placenta previa [RR (95% CI): 2.437 (0.175–33.920); I^2^ = 82.9%, Tau^2^ = 5.6521]. However, significantly increased placenta previa risk after UAE was found in PPH patients [RR (95% CI): 8.739 (1.580–48.341), *p* = 0.013], and when compared with patients without UAE, UAE treatment was associated with a significantly increased risk of placenta previa [RR (95% CI): 10.682 (6.859–16.636), *p* < 0.001; Supplementary Table S3; Supplementary Figure S5].

### 3.5 Trial sequential analysis results

**Figure 5:**
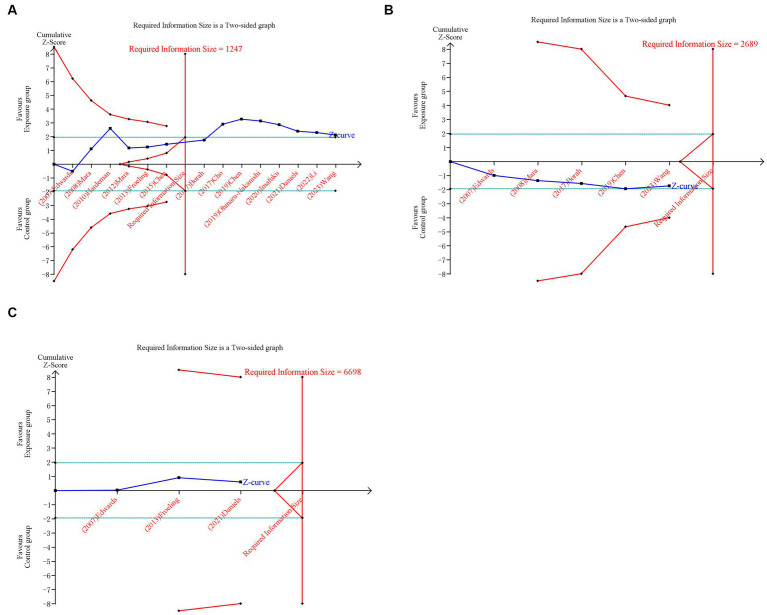
Trial sequential analysis (TSA) of primary outcomes after UAE. **(A)** Pregnancy rate. **(B)** Spontaneous abortion. **(C)** Live birth. Uppermost and lowermost red curves represent trial sequential monitoring boundary lines for benefit and harm, respectively. Horizontal green lines represent the conventional boundaries for statistical significance. Inner red lines represent the futility boundary.

For primary outcomes, only the cumulative Z-curve of pregnancy rate crossed the RIS boundary but failed the trial sequential monitoring boundary, indicating that a relatively definite conclusion of pregnancy rate can be obtained. The cumulative Z-curves of spontaneous abortion and live birth did not pass the RIS boundary or trial sequential monitoring boundary, indicating that the ability to make a definitive conclusion concerning spontaneous abortion and live birth was limited, potentially due to the presence of false positives (Figure 5). For secondary outcomes, the cumulative Z-curves of cesarean section, preterm delivery, and placenta previa crossed the RIS boundary, but the cumulative Z-curves of ectopic pregnancy and PPH neither crossed the RIS boundary nor the trial sequential monitoring boundary, suggesting a relatively definite conclusion of cesarean section, preterm delivery, and placenta previa can be obtained (Supplementary Figure S6).

### 3.6 Sensitivity analysis and publication bias

Sensitivity analysis and publication bias test were conducted for the pooled results of pregnancy rate, cesarean section, and preterm delivery, which included ≥7 studies. In the process of conducting a sensitivity analysis, the pooled RRs and their corresponding 95% CIs were computed, excluding individual studies in turn to evaluate the potential effect of single studies on the overall results. The sensitivity analysis revealed that Cho’s and Imafuku’s study may be the cause of high heterogeneity of cesarean section and preterm delivery, respectively (Supplementary Figure S7). Begg’s test and Egger’s test results showed that no significant publication bias existed in pregnancy rate and preterm delivery (all *p* > 0.05). Egger’s test results indicated the existence of publication bias in cesarean section (*p* = 0.039). Furthermore, the trim-and-fill method was used to adjust for publication bias. After adjusting for publication bias, a comparison of the adjusted results with the previous ones revealed no substantial differences, suggesting that these results are still reliable. The funnel plot is shown in Supplementary Figure S8.

## 4 Discussion

In recent years, the utilization of UAE has gained widespread recognition as an effective treatment for various benign gynecological and obstetric conditions, including PPH, placenta previa, UFs, and CSP (1, 41–43). However, concerns have been raised regarding the potential adverse effects of UAE on postoperative ovarian function and fertility, thereby limiting its applicability to patients desiring future fertility (2, 44). The preservation of fertility and pregnancy has emerged as a crucial consideration due to the compromised blood supply associated with the UAE. In this context, the compromise of both uterine and ovarian blood flow assumes significance, as achieving uterine arterial embolization, the primary objective of UAE, may threaten future pregnancy. Furthermore, the possibility of anastomoses between uterine and ovarian arteries raises concerns that the embolization agent could inadvertently enter the ovarian artery, leading to impaired ovarian function and subsequent infertility (45). Additionally, the radiation dose administered in UAE has the potential to detrimentally affect normal ovarian function and fertility (46). With significant improvements in the materials, size, and morphology of the embolic agents used in UAE, as well as the use of anti-Mullerian hormone (AMH), a new indicator for evaluating ovarian function, it has been found in recent studies that that UAE has no significant effect on ovarian reserve function (47, 48). To provide valuable insights into the standardized management of gynecological and obstetric conditions in women with fertility aspirations, this study aimed to explore the influence of UAE on pregnancy rate and outcomes in women of reproductive age.

Our analysis showed that in the total population, female patients had a significantly lower pregnancy rate after UAE treatment compared with the control group. However, the results of the subgroup analysis suggested that UAE did not play a role in reducing pregnancy rates in patients with PPH, CSP, UFs, or other conditions. Furthermore, UAE was not suggested to decrease postoperative pregnancy rate, either compared with myomectomy, HIFU, non-use of UAE, or other treatment modalities. The diminished pregnancy rate observed following UAE primarily stems from the impact on ovarian blood supply and endometrial function. Infertility can arise from various factors, including fallopian tube obstruction, compromised endometrial receptivity due to structural distortions, alterations in endometrial development, and hormonal imbalances (49). Furthermore, unshielded radiation during the procedure in UAE may jeopardize the uterus and ovaries, compromising fertility (50). Radiation exposure severely alters ovarian performance, manifesting as the shrinkage of follicles and a decrease in follicle reserves. This accelerates the innate depletion of follicle count, resulting in compromised ovarian hormone synthesis, uterine malfunction from insufficient estrogen, early-onset menopause, and infertility (51–53). Beyond impacting the ovaries, radiation exerts adverse effects on the uterus. This may manifest as placental anomalies (e.g., placenta accreta), fetal malposition, premature delivery, and even, albeit rarely, uterine rupture (46, 54). Nevertheless, we cannot generalize the overall analysis results to the whole population. The reasons need to be elucidated. The outcome of pregnancy rate revealed substantial heterogeneity between studies, impeding precise predictions regarding obstetric outcomes. Moreover, the overall analysis included studies with different patient origins and treatments of the control group, alongside discrepancies in study design, thereby contributing to significant heterogeneity between studies. Although subgroup analyses were conducted, further subgroup analyses based on the same patients and control group treatments were unfeasible due to the limited number of included studies. Mothers’ age and follow-up time may also be sources of heterogeneity in the overall analysis.

A previous review posited that the UAE serves as a safe alternative to surgery for women who do not desire to preserve fertility or for cases with elevated surgical risk (55). A subsequent review that did not include RCTs noted that UAE is an alternative treatment to myomectomy for women aspiring to conceive (56). Myomectomy, encompassing hysteroscopic, laparoscopic, abdominal, or transvaginal approaches, stands as a widely employed procedure for leiomyoma removal (57). For UF patients or compared with myomectomy, a significantly decreased risk of preterm delivery after UAE was observed in our subgroup analysis; no association was found between UAE and an increased risk of spontaneous abortion, live birth, ectopic pregnancy, cesarean section, PPH, and placenta previa. Mohan et al. conducted a comprehensive review of 21 studies and concluded that the impact of UAE on fertility remains uncertain, given the influential confounding factors of age and fibroid type in fertility evaluations (58). The Cochrane Review indicated limited evidence suggesting the potential benefits of myomectomy over UAE in improving fertility outcomes. However, this evidence was not substantial, necessitating further investigation (59). Consequently, in the absence of definitive data guiding patient advice, the SIR made the following recommendations (10): (i) For patients with a history of myomectomy, high-quality studies have not reported reproductive outcomes. Given the challenges associated with repeated surgery, embolization might be a preferable option. (ii) For patients deemed unsuitable for surgery due to factors such as complications, physical disposition, or the location or extent of leiomyomas, uterine embolization presents a viable option for those aspiring to conceive. CSP, a long-term complication of cesarean delivery, has exhibited a persistent upward trend (60). The gestational sac in CSP patients primarily resides within cicatrix tissue, characterized by a thin muscular layer. Consequently, arresting bleeding through the contraction of this delicate muscle layer poses a challenge. During the separation of the gestational sac or placental tissue, the previous incision site may rupture, leading to uncontrollable bleeding, thereby further compromising women’s wellbeing. UAE represents a treatment modality capable of promptly halting bleeding and averting massive hemorrhage (61, 62). Our subgroup analysis showed that UAE decreased the risk of cesarean section and was not associated with spontaneous abortion in patients with CSP. However, this subgroup analysis was limited by the fact that the results were pooled from only 2 studies (Chen et al. and Wang et al.) and that the patients in both studies were from China. It is not convincing to generalize the pooled results to the whole population. In addition, the control treatments in these two studies were HIFU. Thus, these findings offer certain reference values for UAE and HIFU treatment in CSP patients. More relevant research is needed to refine and validate the results. The pooled results of 2 studies indicated that UAE increased the risk of placenta previa and was not associated with cesarean section and preterm delivery in patients with PPH. Salomon et al. reported 4\u00B0cases of successful deliveries following previous UAE, yet all experienced recurrent PPH, necessitating hysterectomy in 2 patients. They postulated that uterine damage resulting from UAE, via an unknown mechanism, might contribute to abnormal placentation, thereby inducing PPH (63).

HIFU, a novel thermal ablation technique, exhibits lower toxicity when compared with alternative ablation methods (64). Compared with HIFU, UAE reduced the risk of cesarean section in the present analysis. The underlying mechanism of cesarean section following UAE remains unclear due to limited available data. Nevertheless, HIFU enables the precise focusing of waves to induce coagulation in targeted fibroids, thereby preserving the integrity of the myometrium and endometrium during ablation (65, 66). In this sense, HIFU ablation holds promise for achieving favorable pregnancy outcomes. In addition, UAE increased the risk of postoperative placenta previa compared with no use of UAE. Nonetheless, the relatively wide 95% CI suggested an instability of the result. Hence, additional studies are needed to further validate and supplement the above results.

Our overall analysis showed that compared with the control, UAE treatment increased the risk of postoperative PPH in the total population. Similar results were shown only in patients with PPH. There is only one RCS comparing UAE vs. control on postoperative PPH in PPH patients; we cannot generalize the results of only one study to the entire population. It is well established that women with a history of PPH are at elevated risk of recurrent PPH (67). Notably, the frequency of PPH demonstrates an increase after UAE therapy for uterine myoma, as compared with cases involving laparoscopic myomectomy, thereby implicating UAE as a potential risk factor for PPH (68). Moreover, independent investigators have also reported a heightened incidence of an abnormally invasive placenta in pregnancies following UAE (69, 70). A subsequent pregnancy following UAE treatment for severe PPH poses an amplified risk of recurrent severe PPH, likely attributed to the presence of an abnormally invasive placenta.

In the present study, several noteworthy points and limitations warrant consideration. First, a significant limitation of the meta-analysis was the presence of substantial heterogeneity. We recognized that numerous factors, including maternal age, fibroid location, and follow-up duration, may exert an effect on pregnancy rate and outcomes, it is regrettable that further sub-analysis based on these factors could not be pursued due to unavailable information in the relevant studies. Second, the cohort studies included did not specify whether the patients participating in the research suffered exclusively from a single disease. For instance, in Imafuku et al.’s study, the causes of PPH included conditions such as uterine atony, abnormally invasive placenta, placenta previa, and UFs. Third, it is ideal to ascertain the pregnancy rate specifically among women who actively desire to conceive. However, the lack of information on desired pregnancies in several studies necessitated an alternative approach. Consequently, the pregnancy rate was calculated by dividing the number of successful pregnancies by the total sample size, resulting in noticeable heterogeneity across studies. Fourth, there was an insufficient number of studies to continue sub-analysis by subgroup 1 and subgroup 2. Nevertheless, despite these limitations, the obtained findings hold direct relevance to daily clinical practice, providing valuable guidance for recommending appropriate treatment options to patients.

## 5 Conclusion

In conclusion, UAE treatment was associated with a lower postoperative pregnancy rate and increased risk of PPH. These findings cannot be explained by subgroup analysis at present. Additionally, when compared with myomectomy, HIFU, and non-use of UAE, UAE was shown to decrease the risk of preterm delivery and cesarean section and increase the risk of placenta previa, respectively. Similar results were found in patients with UFs, CSP, and PPH, respectively. More comparative studies and further subgroup analysis are needed to clarify the association between UAE and pregnancy rate and outcomes.

## Data availability statement

The original contributions presented in the study are included in the article/Supplementary material, further inquiries can be directed to the corresponding authors.

## Author contributions

XY: Formal Analysis, Software, Writing – original draft. LZ: Methodology, Writing – review & editing. GH: Methodology, Validation, Writing – review & editing. XL: Conceptualization, Data curation, Writing – original draft.

## Footnotes

^1^ www.ctu.dk/tsa
