# Supplementary material for: Pregnancy rate and outcomes after uterine artery embolization for women: a systematic review and meta-analysis with trial sequential analysis
Source: Front Med (Lausanne). 2023 Dec 21;10:1283279. doi: 10.3389/fmed.2023.1283279 (PMC10764427; doi:10.3389/fmed.2023.1283279)
Supplement: Supplementary file 1 [file Table_1.DOCX]

| PubMed 1964 |
| --- |
| ((uterine artery embolization) OR UAE) AND ((pregnancy outcome) OR outcomes OR pregnancies OR gestation OR reproduction) AND ((cohort study) OR (concurrent study) OR (cohort analysis) OR (incidence study) OR (follow-up study) OR (longitudinal study) OR (prospective study) OR (retrospective study) OR (randomized clinical trial) OR (randomized controlled clinical trial) OR (randomized controlled trial) OR (controlled clinical trial) OR randomized) |
| Web of Science 1016 |
| #1 TS=((uterine artery embolization) OR UAE) #2 TS=((pregnancy outcome) OR outcomes OR pregnancies OR gestation OR reproduction) #3 TS=((cohort study) OR (concurrent study) OR (cohort analysis) OR (incidence study) OR (follow-up study) OR (longitudinal study) OR (prospective study) OR (retrospective study) OR (randomized clinical trial) OR (randomized controlled clinical trial) OR (randomized controlled trial) OR (controlled clinical trial) OR randomized) #4 #1 AND #2 AND #3 |
| Embase 748 |
| #1 'uterine artery embolization'/exp OR UAE #2 'pregnancy outcome'/exp OR 'outcomes'/exp OR pregnancies OR 'gestation'/exp OR 'reproduction'/exp #3 cohort AND study OR (concurrent AND study) OR (cohort AND analysis) OR (incidence AND study) OR ('follow up' AND study) OR (longitudinal AND study) OR (prospective AND study) OR (retrospective AND study) OR (randomized AND clinical AND trial) OR (randomized AND controlled AND clinical AND trial) OR (randomized AND controlled AND trial) OR (controlled AND clinical AND trial) OR randomized #4 #1 AND #2 AND #3 |
| The Cochrane Library 456 |
| #1 (uterine artery embolization) OR (UAE) (Word variations have been searched) #2 (pregnancy outcome) OR (outcomes) OR (pregnancies) OR (gestation) OR (reproduction) #3 (cohort study) OR (concurrent study) OR (cohort analysis) OR (incidence study) OR (follow-up study) OR (longitudinal study) OR (prospective study) OR (retrospective study) OR (randomized clinical trial) OR (randomized controlled clinical trial) OR (randomized controlled trial) OR (controlled clinical trial) OR randomized #4 #1 AND #2 AND #3 |
