# Supplementary material for: Pregnancy rate and outcomes after uterine artery embolization for women: a systematic review and meta-analysis with trial sequential analysis
Source: Front Med (Lausanne). 2023 Dec 21;10:1283279. doi: 10.3389/fmed.2023.1283279 (PMC10764427; doi:10.3389/fmed.2023.1283279)
Supplement: Supplementary file 2 [file Table_2.DOCX]

| **TABLE S1** The Newcastle-Ottawa quality assessment scale of the included studies. | | | | | | | | | | | | |
| --- | --- | --- | --- | --- | --- | --- | --- | --- | --- | --- | --- | --- |
| Study | Selection | | | |  | Comparability | |  | Assessment of outcome | | | Total score |
|  | Representativeness of exposure arm(s) | Selection of the comparative arm(s) | Origin of exposure source | Demonstration that outcome of interest was not present at start of study |  | Studies controlling the most important factors | Studies controlling the other main factors |  | Assessment of outcome with independency | Adequacy of follow-up length | Lost to follow-up acceptable |  |
|  |  |  |  |  |  |  |  |  |  |  |  |  |
| Hardeman 2010 | 1 | 1 | 1 | 1 |  | 1 | 0 |  | 1 | 1 | 1 | 8 |
| Chen 2015 | 1 | 1 | 1 | 1 |  | 1 | 0 |  | 1 | 1 | 1 | 8 |
| Borah 2017 | 1 | 1 | 1 | 1 |  | 1 | 0 |  | 1 | 1 | 1 | 8 |
| Imafuku 2020 | 1 | 1 | 1 | 1 |  | 1 | 0 |  | 1 | 0 | 1 | 7 |
| Chen 2019 | 1 | 1 | 1 | 1 |  | 1 | 0 |  | 1 | 1 | 1 | 8 |
| Ohmaru-Nakanishi 2019 | 1 | 1 | 1 | 1 |  | 1 | 0 |  | 1 | 0 | 1 | 7 |
| Wang 2023 | 1 | 1 | 1 | 1 |  | 1 | 0 |  | 1 | 1 | 1 | 8 |
| Jitsumori 2020 | 1 | 1 | 1 | 1 |  | 1 | 0 |  | 1 | 0 | 1 | 7 |
| Cho 2017 | 1 | 1 | 1 | 1 |  | 0 | 0 |  | 1 | 0 | 1 | 6 |
| Froeling 2013 | 1 | 1 | 1 | 1 |  | 1 | 0 |  | 1 | 1 | 1 | 8 |
| Li 2022 | 0 | 1 | 1 | 1 |  | 1 | 0 |  | 1 | 1 | 1 | 7 |

| **TABLE S2** Quality analysis of the included studies by modified Jadad scale. | | | | | |  |
| --- | --- | --- | --- | --- | --- | --- |
| Study | Randomization | Randomization concealment | Double blind | Withdrawals and dropouts | Score | Study quality |
| Mara 2008 | 2 | 2 | 0 | 1 | 5 | High |
| Edwards 2007 | 2 | 2 | 0 | 1 | 5 | High |
| Daniels 2021 | 2 | 1 | 0 | 1 | 4 | High |
| Mara 2012 | 0 | 0 | 0 | 1 | 1 | Low |
