# Supplementary material for: Pregnancy rate and outcomes after uterine artery embolization for women: a systematic review and meta-analysis with trial sequential analysis
Source: Front Med (Lausanne). 2023 Dec 21;10:1283279. doi: 10.3389/fmed.2023.1283279 (PMC10764427; doi:10.3389/fmed.2023.1283279)
Supplement: Supplementary file 3 [file Table_3.DOCX]

| TABLE S3 Pooled effect and subgroup analysis of secondary pregnancy outcomes after uterine artery embolization for women. | | | | | | | |
| --- | --- | --- | --- | --- | --- | --- | --- |
| Outcomes and subgroups | Number of study | Meta-analysis | | | | Heterogeneity | |
|  |  | RR | 95% CI | P value | 95% PI | I2, Tau2 | P value |
| **Ectopic pregnancy** | | | | | | | |
| Overall | 3 | 1.218 | 0.420-3.533 | 0.717 | 0.001-1320.509 | 0%, 0 | 0.835 |
| Subgrouped by the origin of participants (Subgroup 1) | | | | | | | |
| UF patients | 3 | 1.218 | 0.420-3.533 | 0.717 | 0.001-1320.509 | 0%, 0 | 0.835 |
| Subgrouped by the treatment of control group (Subgroup 2) | | | | | | | |
| UAE vs. Myomectomy | 2 | 1.017 | 0.302-3.422 | 0.979 | - | 0%, 0 | 0.958 |
| UAE vs. Others | 1 | 2.286 | 0.215-24.316 | 0.493 |  |  |  |
| **Cesarean section** | | | | | | | |
| Overall | 9 | 0.945 | 0.664-1.345 | 0.754 | 0.326-2.741 | 77.1%, 0.1703 | <0.001 |
| Subgrouped by the origin of participants (Subgroup 1) | | | | | | | |
| UF patients | 4 | 0.881 | 0.494-1.570 | 0.667 | 0.101-7.700 | 56.9%, 0.1670 | 0.073 |
| PPH patients | 2 | 1.231 | 0.706-2.144 | 0.464 | - | 77.8%, 0.1300 | 0.034 |
| CSP patients | 2 | 0.693 | 0.481-0.999 | 0.050 | - | 23.9%, 0.0220 | 0.252 |
| Others | 1 | 1.953 | 0.413-9.240 | 0.399 |  |  |  |
| Subgrouped by the treatment of control group (Subgroup 2) | | | | | | | |
| UAE vs. Myomectomy | 3 | 0.574 | 0.184-1.793 | 0.340 | - | 64.2%, 0.6105 | 0.061 |
| UAE vs. Without UAE | 3 | 1.297 | 0.824-2.040 | 0.261 | 0.011-152.267 | 56.7%, 0.0872 | 0.099 |
| UAE vs. HIFU | 2 | 0.693 | 0.481-0.999 | 0.050 | - | 23.9%, 0.0220 | 0.252 |
| UAE vs. Others | 1 | 1.371 | 0.795-2.367 | 0.257 |  |  |  |
| **Preterm delivery** | | | | | | | |
| Overall | 7 | 0.632 | 0.356-1.121 | 0.117 | 0.326-1.609 | 0%, 0 | 0.456 |
| Subgrouped by the origin of participants (Subgroup 1) | | | | | | | |
| UF patients | 3 | 0.326 | 0.128-0.831 | 0.019 | 0.001-176.960 | 0%, 0 | 0.604 |
| PPH patients | 2 | 1.284 | 0.496-3.326 | 0.607 | - | 0%, 0 | 0.339 |
| CSP patients | 1 | 1.015 | 0.147-6.997 | 0.988 |  |  |  |
| Others | 1 | 0.781 | 0.118-5.165 | 0.798 |  |  |  |
| Subgrouped by the treatment of control group (Subgroup 2) | | | | | | | |
| UAE vs. Myomectomy | 2 | 0.296 | 0.106-0.826 | 0.020 | - | 0%, 0 | 0.360 |
| UAE vs. Without UAE | 3 | 1.153 | 0.495-2.686 | 0.742 | 0.004-326.890 | 0%, 0 | 0.560 |
| UAE vs. HIFU | 1 | 1.015 | 0.147-6.997 | 0.988 |  |  |  |
| UAE vs. Others | 1 | 0.571 | 0.054-6.079 | 0.643 |  |  |  |
| **Postpartum hemorrhage** | | | | | | | |
| Overall | 4 | 3.182 | 1.319-7.675 | 0.010 | 0.474-22.089 | 0%, 0 | 0.931 |
| Subgrouped by the origin of participants (Subgroup 1) | | | | | | | |
| UF patients | 2 | 3.453 | 0.862-13.830 | 0.080 | - | 0%, 0 | 0.968 |
| PPH patients | 1 | 3.810 | 1.053-13.787 | 0.042 |  |  |  |
| Others | 1 | 1.563 | 0.150-16.265 | 0.709 |  |  |  |
| Subgrouped by the treatment of control group (Subgroup 2) | | | | | | | |
| UAE vs. Myomectomy | 2 | 3.453 | 0.862-13.830 | 0.080 | - | 0%, 0 | 0.968 |
| UAE vs. Without UAE | 2 | 2.954 | 0.971-8.991 | 0.057 | - | 0%, 0 | 0.510 |
| **Placenta previa** | | | | | | | |
| Overall | 4 | 2.437 | 0.175-33.920 | 0.507 | - | 82.9%, 5.6521 | 0.001 |
| Subgrouped by the origin of participants (Subgroup 1) | | | | | | | |
| UF patients | 1 | 0.228 | 0.011-4.617 | 0.335 |  |  |  |
| PPH patients | 2 | 8.739 | 1.580-48.341 | 0.013 | - | 50.4%, 0.9887 | 0.156 |
| Others | 1 | 2.364 | 0.100-55.661 | 0.594 |  |  |  |
| Subgrouped by the treatment of control group (Subgroup 2) | | | | | | | |
| UAE vs. Without UAE | 3 | 10.682 | 6.859-16.636 | <0.001 | - | 48.0%, 1.0423 | 0.146 |
| UAE vs. Others | 1 | 0.228 | 0.011-4.617 | 0.335 |  |  |  |
