# Supplementary material for: Pregnancy rate and outcomes after uterine artery embolization for women: a systematic review and meta-analysis with trial sequential analysis
Source: Front Med (Lausanne). 2023 Dec 21;10:1283279. doi: 10.3389/fmed.2023.1283279 (PMC10764427; doi:10.3389/fmed.2023.1283279)

**Figure S1** Forest plot of secondary outcome of ectopic pregnancy after UAE. (A) Overall analysis. (B) Analysis by subgroup 1. (C) Analysis by subgroup 2.


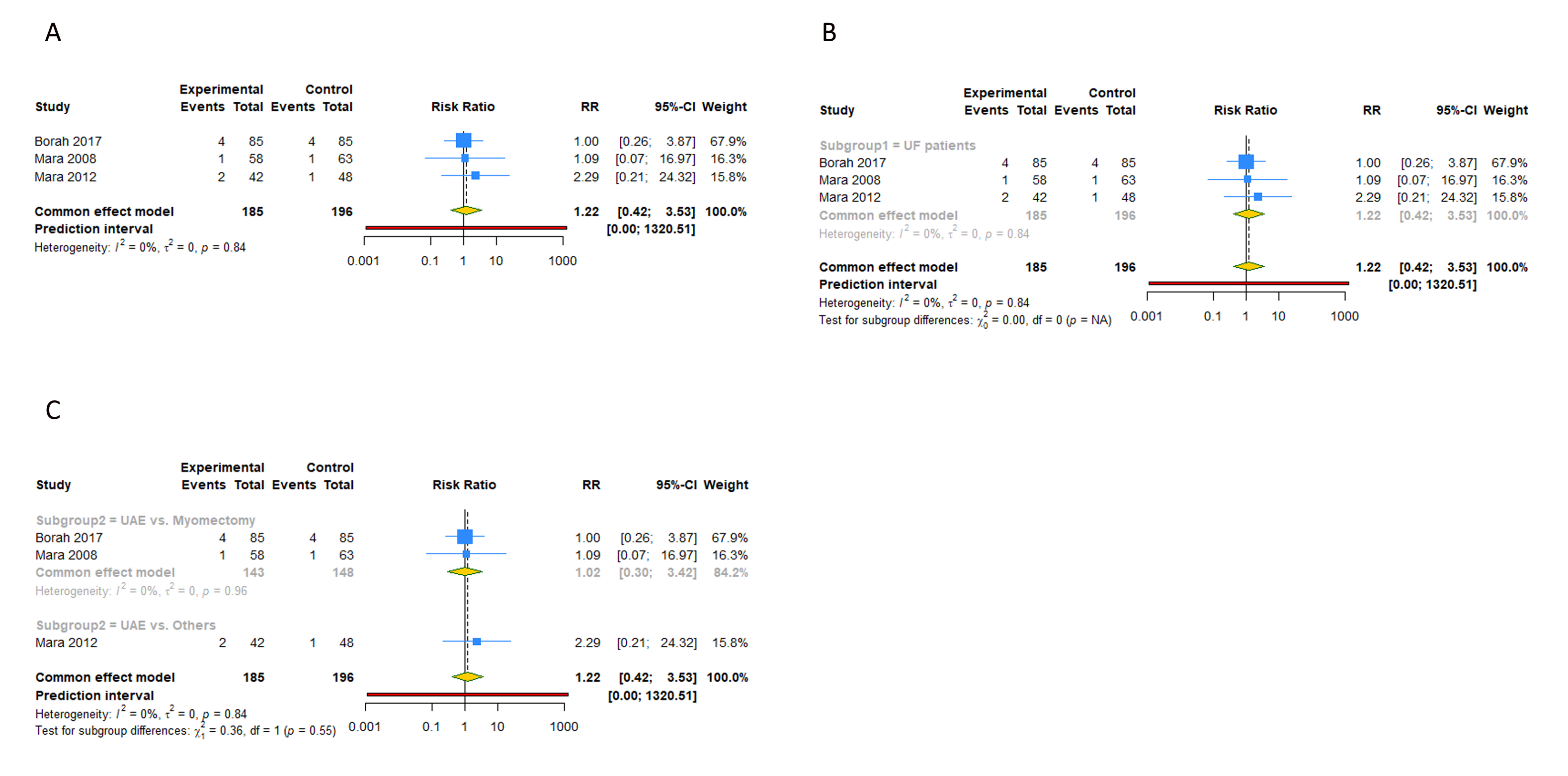


**Figure S2** Forest plot of secondary outcome of cesarean section after UAE. (A) Overall analysis. (B) Analysis by subgroup 1. (C) Analysis by subgroup 2.


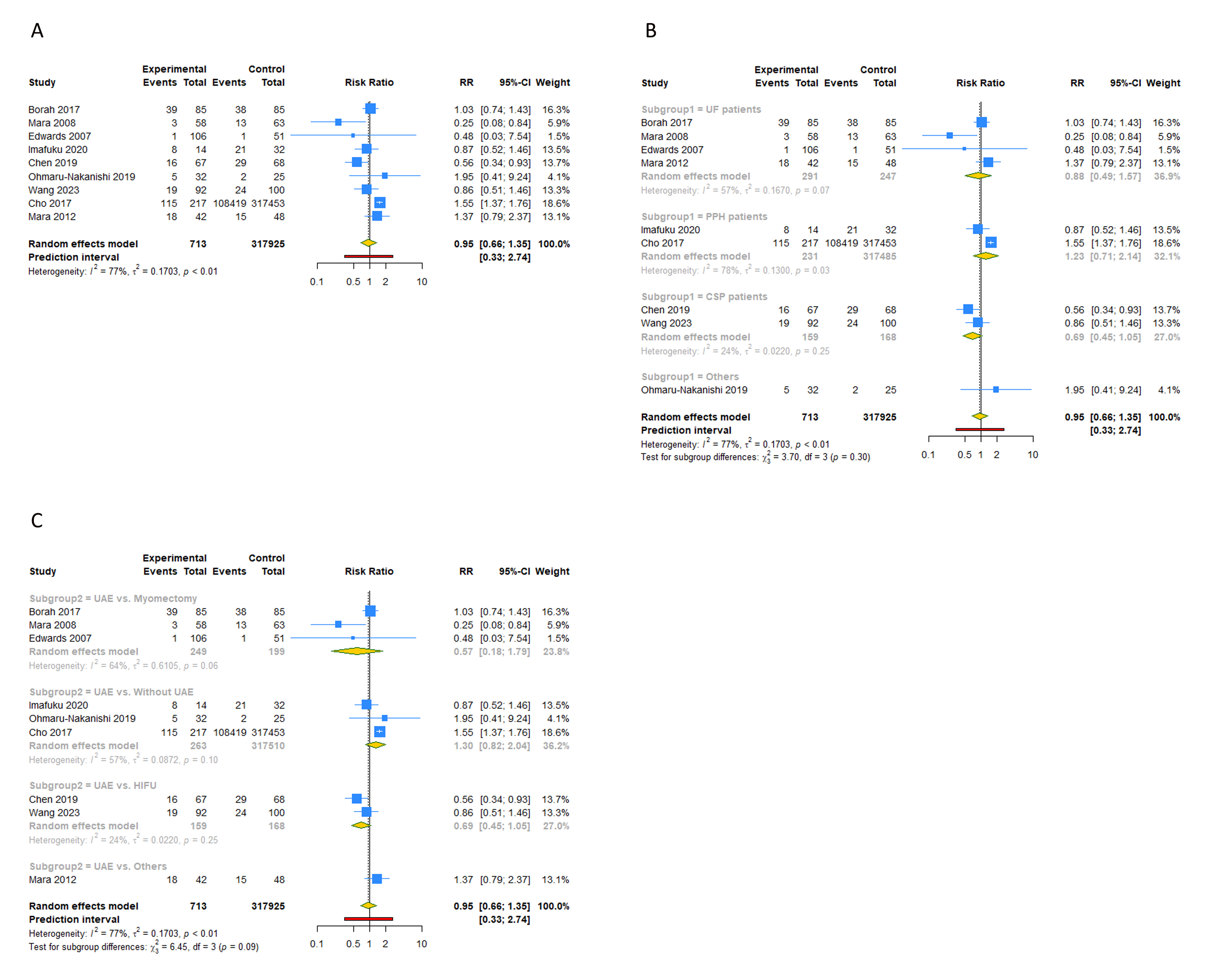


**Figure S3** Forest plot of secondary outcome of preterm delivery after UAE. (A) Overall analysis. (B) Analysis by subgroup 1. (C) Analysis by subgroup 2.


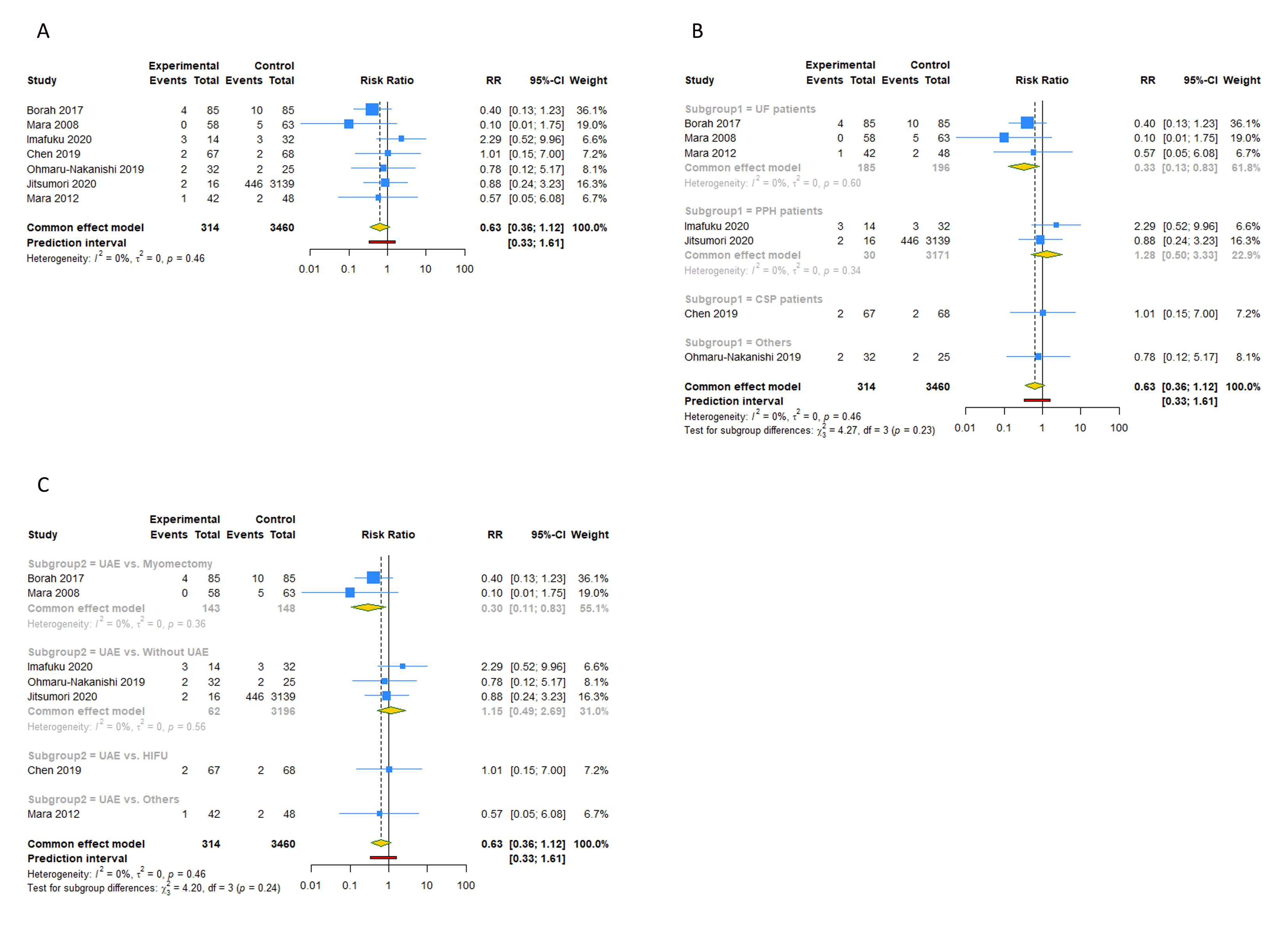


**Figure S4** Forest plot of secondary outcome of postpartum hemorrhage after UAE. (A) Overall analysis. (B) Analysis by subgroup 1. (C) Analysis by subgroup 2.


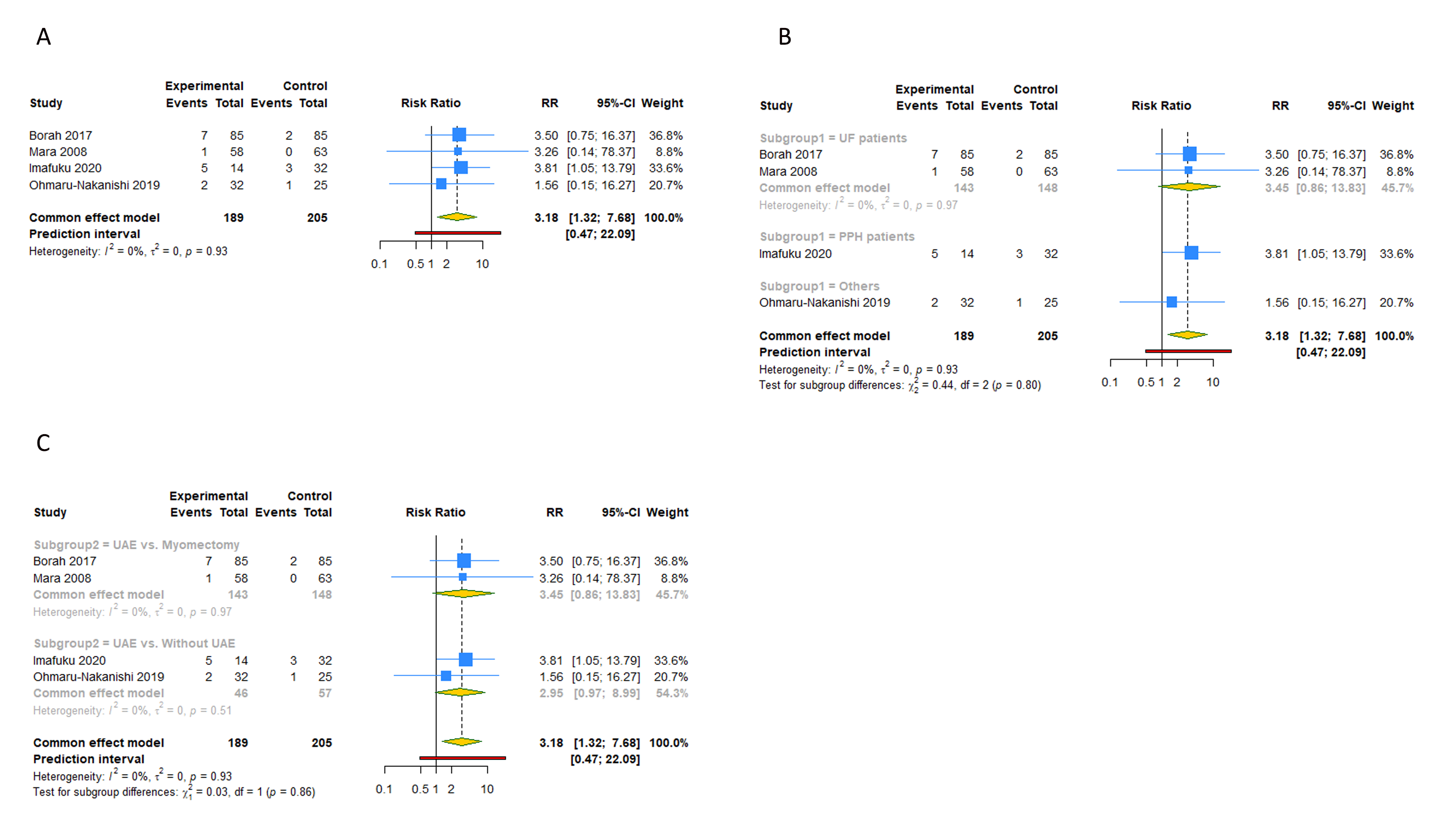


**Figure S5** Forest plot of secondary outcome of placenta previa after UAE. (A) Overall analysis. (B) Analysis by subgroup 1. (C) Analysis by subgroup 2.


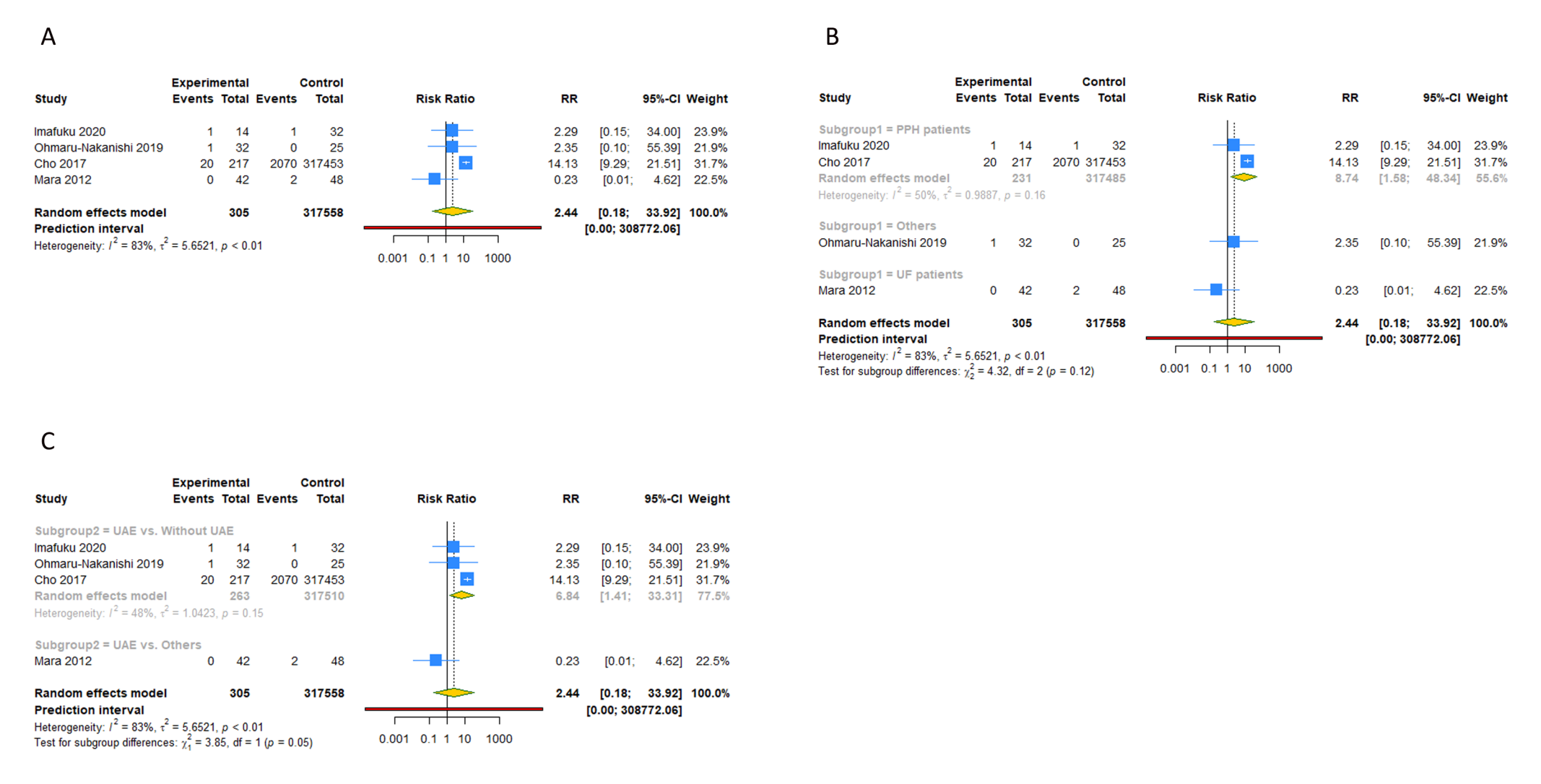


**Figure S6** Trial sequential analysis (TSA) of secondary outcomes after UAE. (A) Ectopic pregnancy. (B) Cesarean section. (C) Preterm delivery. (D) Postpartum hemorrhage. (E) Placenta previa. Uppermost and lowermost red curves represent trial sequential monitoring boundary lines for benefit and harm, respectively. Horizontal green lines represent the conventional boundaries for statistical significance. Inner red lines represent the futility boundary.


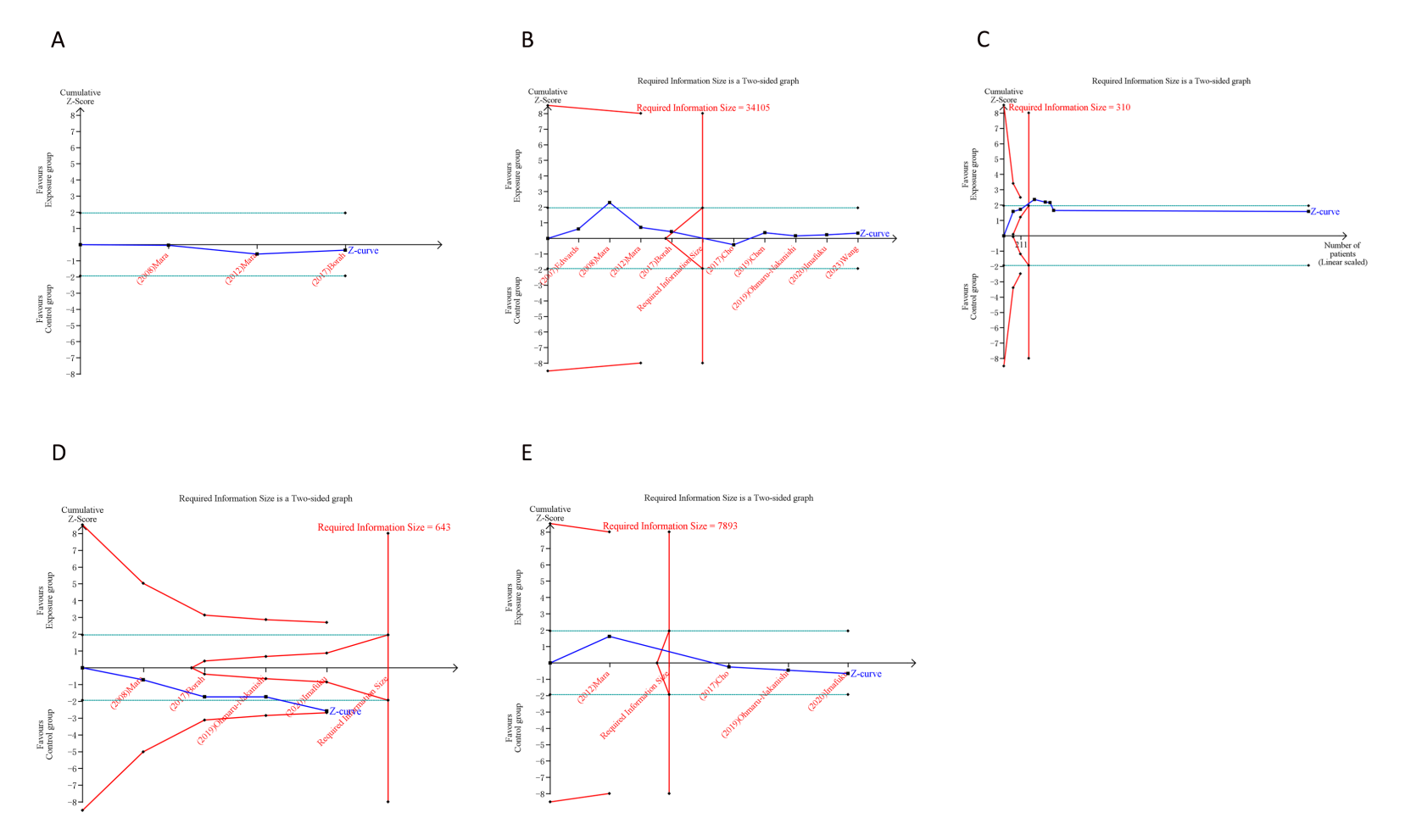


**Figure S7** Sensitivity analysis on the pregnancy rate and outcomes after UEA. (A) Pregnancy rate. (B) Cesarean section. (C) Preterm delivery.


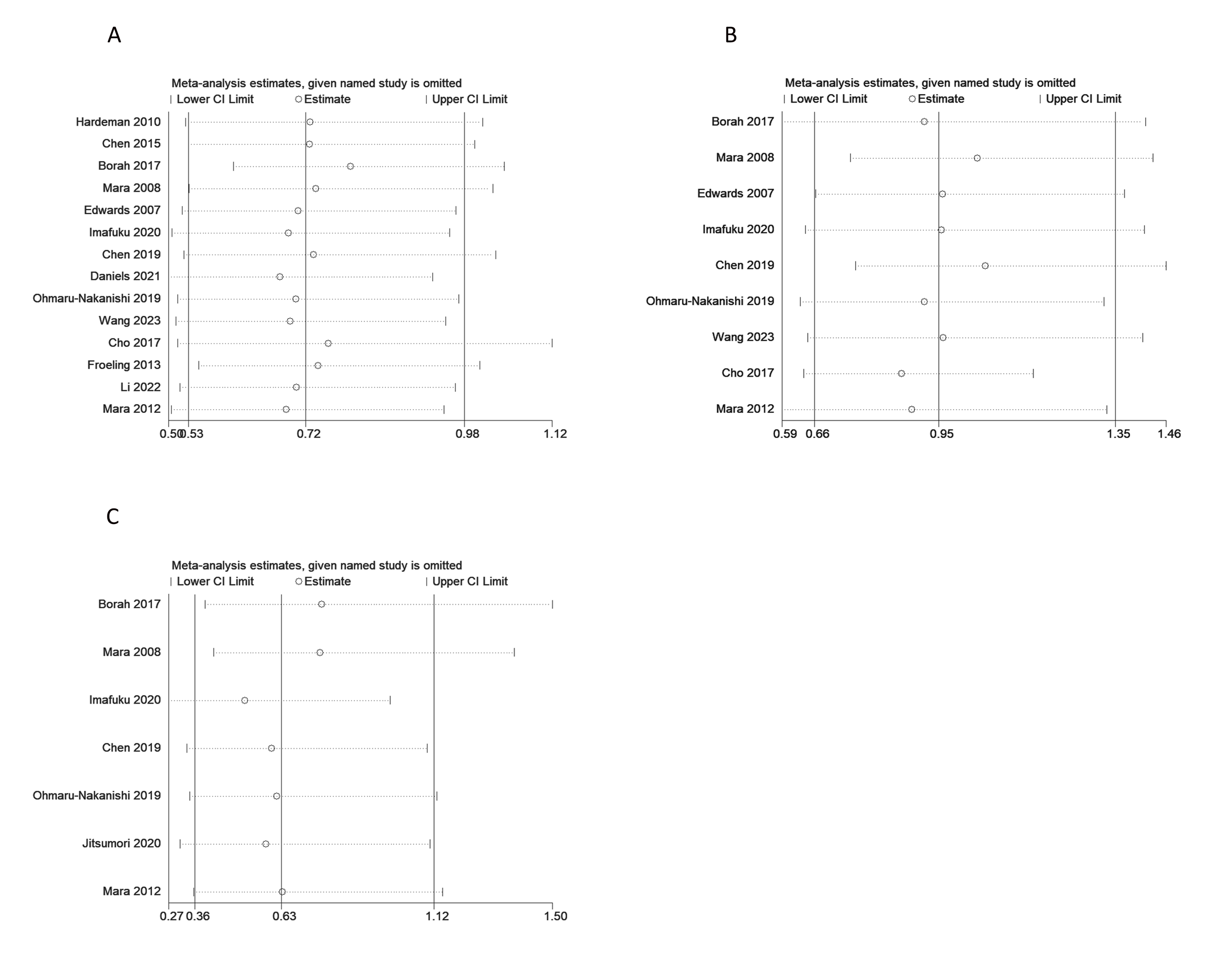


**Figure S8** Funnel plot of the pregnancy rate and outcomes after UEA. (A) Pregnancy rate. (B) Cesarean section. (C) Preterm delivery.


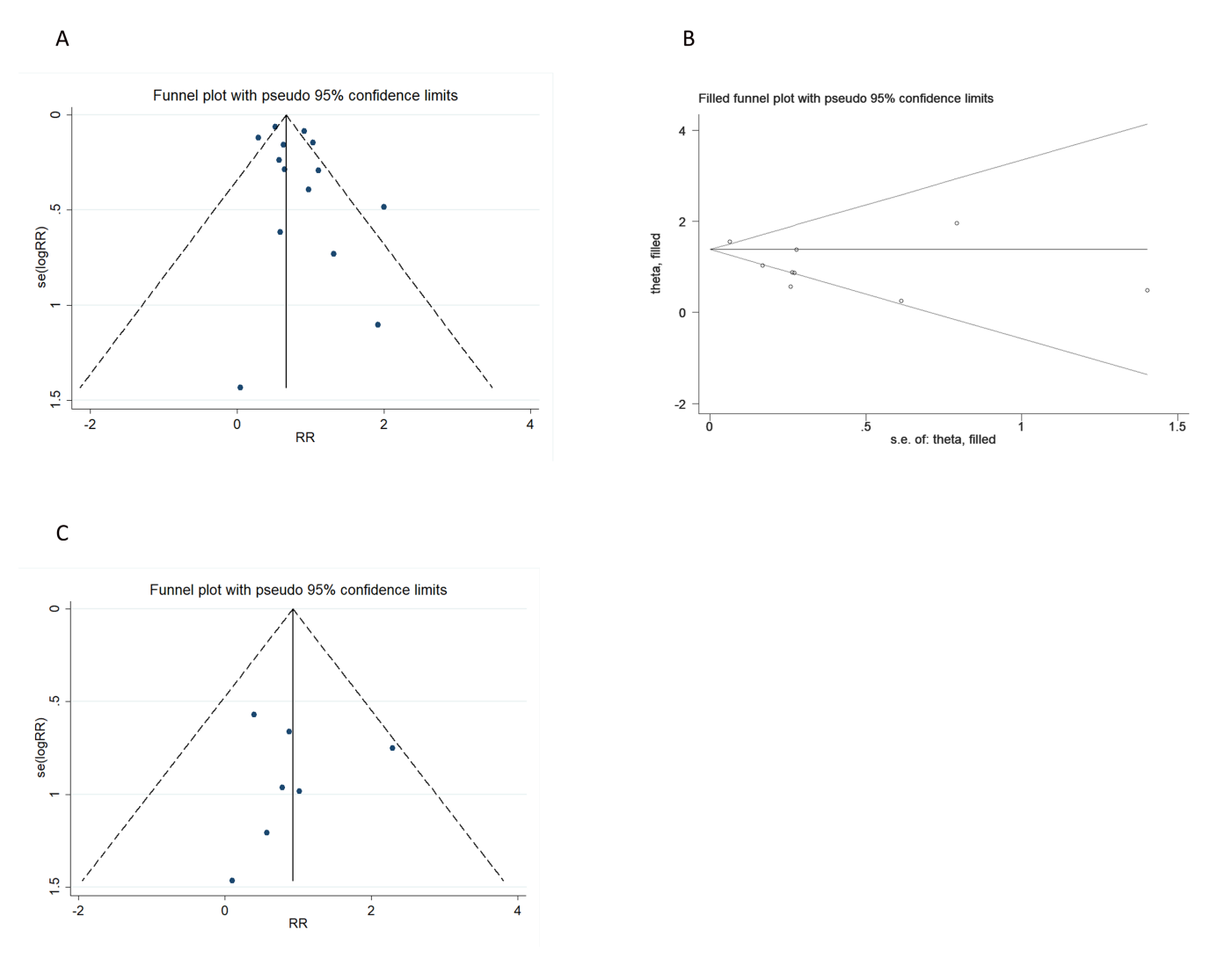

Supplement: Supplementary file 4 [file Data_Sheet_1.docx]
